# Veterans’ Donations of Research Incentives to Fellow Veterans

**DOI:** 10.1001/jamanetworkopen.2025.20307

**Published:** 2025-07-14

**Authors:** Donna M. Zulman, Mayuree Rao, Cindie Slightam, Liberty Greene, Matthew L. Maciejewski

**Affiliations:** 1 Center for Innovation to Implementation, Veterans Affairs Palo Alto Health Care System, Menlo Park, California; 2 Division of Primary Care and Population Health, Stanford University School of Medicine, Stanford, California; 3 Seattle-Denver Center of Innovation for Veteran-Centered and Value-Driven Care, VA Puget Sound Health Care System, Seattle, Washington; 4 Department of Medicine, University of Washington, Seattle; 5 Center of Innovation to Accelerate Discovery and Practice Transformation, Durham Veterans Affairs Health Care System, Durham, North Carolina; 6 Department of Population Health Sciences, Duke University, Durham, North Carolina; 7 Duke-Margolis Center for Health Policy, Duke University, Durham, North Carolina

## Abstract

This survey study examines using monetary donations to veteran-serving organizations as an incentive for research participation among US veterans.

## Introduction

After serving their country, US veterans often continue public service by volunteering to participate in Veterans Affairs (VA) research studies. Their contributions have been instrumental to numerous scientific advancements, including medical treatments for posttraumatic stress disorder, development of the implantable cardiac pacemaker, and the world’s largest genomic biorepository, the Million Veteran Program.^1^

Research studies sometimes offer financial incentives to boost participation rates, but the impact can be modest.^2^ Given that many veterans are motivated to participate in research that helps fellow veterans, we evaluated VA research participant preferences for receiving a monetary incentive or donating an equal amount to a veteran-focused service organization.

## Methods

During March through June 2024, we conducted a social risks survey on a national sample of veterans, following the AAPOR guideline for survey studies. During survey development, a veteran engagement group suggested that we offer respondents the option of donating their monetary incentive to an organization that supports veterans.

The survey was administered via web and mail to a stratified national sample of 13 510 veterans who received VA primary care and/or mental health care in the previous year, with stratification by age, race and ethnicity, and sex and oversampling of small strata. After the survey, respondents were offered $10, which they could receive as cash or an Amazon e-gift card or donate to Disabled American Veterans. The survey included a 2-item measure of financial strain that queried respondents’ general financial situation and difficulty affording basic needs^3,4^ and measures of self-reported race and ethnicity to enable more precise, veteran-centered characterization of these variables than is available in the VA health record. We calculated the unweighted proportion of respondents who elected to donate the monetary incentive and compared donation rates between those who did and did not endorse financial strain using a χ^2^ test, with 2-sided *P* < .05 considered significant. We used Stata, version 18 to conduct analyses. The VA Central Institutional Review Board approved this study and waived informed consent as survey completion was considered consent to participate in the study.

## Results

Among 3430 respondents (response rate, 25%), mean (SD) age was 61 (15.2) years; 1446 (42.2%) were women, 901 (26.3%) were Black, 834 (24.3%) were Hispanic, and 1775 (51.7%) experienced financial strain. Overall, 1720 respondents (50.2%) elected to donate their monetary incentive, 1615 (47.1%) elected to receive an e-gift card or cash, and 95 (2.8%) did not select an option. Respondents experiencing financial strain were less likely to donate their incentive than those not experiencing financial strain (690 [38.9%] vs 991 of 1590 [62.3%]; *P* < .001).

## Discussion

Over half of the participants in this study chose to donate their research incentive, resulting in a $17 200 donation to Disabled American Veterans. This finding suggests that for many veterans, participation in this study was likely driven by a desire to contribute to research supporting fellow veterans.

Monetary incentives have historically affected survey response rates more than donation opportunities.^2^ Guided by veteran input, we opted to offer respondents a choice. Veterans might have selected the donation option because the monetary incentive was modest; however, even among individuals facing financial strain, over one-third of participants chose to donate. Future research should examine whether similar patterns are observed in the general population.

The generosity of study participants reflects an ethos of service common among veterans and rooted in military experience. Veterans report that they are motivated to participate in research, veteran engagement groups, and national peer reviews to contribute to efforts that benefit other veterans.^5,6^ Although we report unweighted results from a survey with a 25% response rate, the selection of the donation option might reflect preferences among many VA research participants because of a commitment to serving others who have served. Just as the scientific advancements achieved through veterans’ engagement in research benefit the broader population,^1^ the actions of veterans in this study might inspire VA and non-VA researchers to offer donation as an incentive in future studies.

## Notes

Supplement.
Data Sharing Statement
