# Supplementary material for: Veterans’ Donations of Research Incentives to Fellow Veterans
Source: JAMA Netw Open. 2025 Jul 14;8(7):e2520307. doi: 10.1001/jamanetworkopen.2025.20307 (PMC12260992; doi:10.1001/jamanetworkopen.2025.20307)
Supplement: Supplement. — Data Sharing Statement [file jamanetwopen-e2520307-s001.pdf]

Data Sharing Statement

Zulman. Veterans' Donations of Research Incentives to Fellow Veterans. *JAMA Netw Open*. Published July 14, 2025. doi:10.1001/jamanetworkopen.2025.20307

Data
Data available: No

Additional Information
Explanation for why data not available: We can share a data dictionary, but the Department of Veterans Affairs does not permit data sharing
